# Mechanisms of Senescence and Anti-Senescence Strategies in the Skin

**DOI:** 10.3390/biology13090647

**Published:** 2024-08-23

**Authors:** Evangelia Konstantinou, Eliane Longange, Gürkan Kaya

**Affiliations:** 1 Department of Medicine, University of Geneva, Rue Michel-Servet 1, CH-1206 Geneva, Switzerland; evangelia.konstantinou@unige.ch (E.K.); eliane.longange@unige.ch (E.L.); 2 Departments of Dermatology and Clinical Pathology, Geneva University Hospitals, Rue Gabrielle Perret-Gentil 4, CH-1205 Geneva, Switzerland

**Keywords:** skin aging, cellular senescence, SASP, p16INK4a, senolytics, senomorphics, senotherapy

## Abstract

**Simple Summary:**

The skin is the outermost barrier of the human body and consists of different layers and cell types. Several environmental and genetic factors can induce skin aging and age-related diseases. One of the main problems in skin aging is that senescent cells are accumulated and secrete factors, which can induce senescence in other tissues. Many researchers are trying to identify treatment modalities (known as senotherapies) to eliminate the senescent cells and reverse the aging process for chronic age-related diseases. The aim of this study is to address the mechanisms that induce senescence and the molecules with potential HAFi effects that are currently investigated for skin aging. Further studies should be conducted to elucidate all the effects of current senotherapies on the skin and other organs. Current data suggest that ongoing research projects in the field may lead to the discovery of new effective anti-senescence strategies in the skin.

**Abstract:**

The skin is the layer of tissue that covers the largest part of the body in vertebrates, and its main function is to act as a protective barrier against external environmental factors, such as microorganisms, ultraviolet light and mechanical damage. Due to its important function, investigating the factors that lead to skin aging and age-related diseases, as well as understanding the biology of this process, is of high importance. Indeed, it has been reported that several external and internal stressors contribute to skin aging, similar to the aging of other tissues. Moreover, during aging, senescent cells accumulate in the skin and express senescence-associated factors, which act in a paracrine manner on neighboring healthy cells and tissues. In this review, we will present the factors that lead to skin aging and cellular senescence, as well as ways to study senescence in vitro and in vivo. We will further discuss the adverse effects of the accumulation of chronic senescent cells and therapeutic agents and tools to selectively target and eliminate them.

## 1. Introduction

### 1.1. Skin Morphology

The skin is an organ consisting of a variety of cells and layers that form a single structure to protect against, and communicate with, the external environment. This dynamic organ is involved in many vital processes, crucial to the health of vertebrates, such as the regulation of body temperature, fluid balance, synthesis of vitamins and hormones and monitoring of immunological responses. The skin is organized into three distinct layers: epidermis, dermis and hypodermis. These will be described in more detail below [1].

#### 1.1.1. Epidermis

The epidermis is the outermost layer of the skin, which forms a barrier against toxins, pathogens and dehydration. Morphologically, the epidermis is a stratified squamous epithelium with constant renewal capability, mainly composed of epidermal cells called keratinocytes (95%). Other cell types present in the epidermis are melanocytes (3%), Langerhans (2%) and Merkel cells (0.5%). There are up to five morphologically distinct sub-layers (strata) of the epidermis, from the innermost to the outermost layer: stratum basale, stratum spinosum, stratum granulosum, stratum lucidum and stratum corneum [2,3]. 

##### Cell Populations of the Epidermis

The majority of epidermal cells are keratinocytes, which undergo cellular differentiation to form different epidermal strata. During their maturation process, keratinocytes undergo changes in their appearance and in the cytoskeletal organization. Keratinocytes play an important supporting role in epidermal structure, contributing to cell viability and signaling pathways such as protein synthesis, cell and epithelial activity, etc. [1,2].

Melanocytes are cells that produce melanin and are located in the stratum basale sub-layer. Melanin plays a role in protection against the harmful effects of UV rays by absorbing them while also determining skin color. In more detail, melanin production is regulated by the melanocyte-stimulating hormone and takes place in melanosomes, which are synthesized from dendritic melanocytes. After its production, melanin is transported via dendrites to keratinocytes and protects the nucleus from UV light, acting as a filter that absorbs the majority of UV [1,2].

Langerhans cells are immune sentinels and are most prominent in the stratum spinosum. These cells can also adopt a dendritic-like phenotype, migrate, and interact with naive T-cells, activating an immune response [2].

Merkel cells are located in the basal layer of the epidermis and in the epithelial sheath of hair follicles. They are connected with the nerve endings and function as sensory receptors [1,2]. 

#### 1.1.2. Dermal–Epidermal Junction

The epidermis and dermis are closely linked by the dermal–epidermal junction. This basal membrane is formed by the keratinocytes of the basal layer and the fibroblasts of the dermis. The dermal–epidermal junction functions as a semi-permeable barrier, controlling the exchange of oxygen, nutrients and waste molecules between the dermis and epidermis [1,2]. Furthermore, the dermal–epidermal junction has an undulating pattern due to the rete ridges, which are epithelial projections that extend the epidermis more deeply into the dermis and enhance their connectivity. These structures increase the surface of the dermal–epidermal junction, improve the mechanical properties of the skin, maintain skin homeostasis and act as protective niches for keratinocyte stem cells. The dimensions of rete ridges are different according to the body site and age and are increased during inflammatory skin diseases [4,5,6].

#### 1.1.3. Dermis

The dermis is a resistant yet flexible layer that supports the epidermis and links it with the hypodermis. It also plays a role in thermoregulation, oxygen supply to the epidermis and elimination of epidermal waste. The dermis is composed of two distinct layers. The upper layer, named papillary dermis, is located below the dermal–epidermal junction and consists mainly of loosely packed collagen and elastin fibers forming a spongy structure. The lower layer, the reticular dermis, is a thicker layer, with denser collagen and elastic fibers [1]. The major cell type in the dermis is dermal fibroblasts, which secrete proteins of the extracellular matrix, such as collagens, elastin and proteoglycans. The main collagen types found in the dermis are as follows: type I and type III. On the other hand, elastin and fibrillin microfibrils are the main types of elastic fibers present in the dermis. Additionally, two more subtypes of elastic fibers can be found in the dermis: elaunin fibers, which are located near the junction of papillary and reticular dermis, and oxytalan fibers, which are in the papillary dermis [7]. The dermis also contains other appendages, such as hair follicles, sweat glands, sebaceous glands, lymphatic vessels, nerves and blood vessels [1,2]. 

#### 1.1.4. Hypodermis

The hypodermis, or subcutaneous tissue, is the deepest layer of the skin, anchoring the dermis to the underlying muscles. This tissue consists mainly of adipocytes (fat cells), connective tissue (interlobular septa) and larger nerves and blood vessels. The hypodermis insulates the body against cold and heat, provides physical protection and serves as an energy reserve [1,2]. 

### 1.2. Skin Aging 

Skin aging is a complex and multifaceted process induced by intrinsic and extrinsic factors that promote biochemical and structural changes to the skin and surrounding tissues. The combination of internal and external factors that induce aging, as well as the human body’s response to these factors, is known as the “skin aging exposome” [8]. Intrinsic aging is induced by chronological aging and is mainly associated with cellular senescence caused by endogenous damage and genetic alterations, while extrinsic aging is driven by environmental factors. Given the impact that skin aging may have on people’s social lifestyle and health, extensive research is required to obtain mechanistic insights into the aging process and to develop anti-aging treatments. In this review, we will provide an overview of the mechanisms of skin aging and tools to combat senescent cells in the skin [9].

#### 1.2.1. Intrinsic Aging

Chronologically dependent skin aging, known as intrinsic aging, is mainly characterized by skin thinning, fine lines and the inability of the skin to repair itself [10,11]. Intrinsic aging is a slow process, and variations between different genders, ethnicities and geographical populations may exist [12]. The main characteristic of intrinsic aging is the alteration of several dermal components, most prominently a reduction in the extracellular matrix (ECM) elements, such as collagens, elastin, glycosaminoglycans, etc., which results in a decline in skin thickness [13,14]. Furthermore, over a lifetime, the flexibility of the skin decreases, and changes that occur in the connective tissue can result in reduced strength and elasticity of the skin, a phenomenon known as elastosis. Changes in skin elasticity can begin early in life and progress during aging [9,10].

Moreover, the continuous production of reactive oxygen species (ROS), either by mitochondrial leakage or inflammations, results in endogenous damage to cellular components such as membranes and enzymes and accelerates telomere shortening, thereby promoting intrinsic aging [13,15,16]. It is well reported that telomeres are repetitive sequences of DNA at the ends of linear chromosomes; their length is tissue-specific and shortens between germline and somatic tissues. Studies have shown that telomeres shorten as normal human fibroblasts grow and also in skin samples from older people, compared to younger [17]. During aging, the lack of telomerase activity and the inability of DNA polymerases to replicate the telomere C-rich strand lagging result in shorter telomeres. As a result, they are recognized as DNA damage, which triggers a DNA damage response (DDR) and cellular senescence [18,19]. The p53-p21 pathway plays an important role in telomere-induced senescence, as DDR is induced by p53, which in turn positively regulates p21 [20].

During aging, a decrease in the levels of different growth factors, signaling molecules, hormones and their receptors occurs, resulting in impairment of several skin functions [21,22,23]. An important example is impaired wound healing. As people age, the proliferative rate of keratinocytes, extracellular matrix synthesis and angiogenesis decrease compared to younger, healthy individuals [23]. On the other hand, the expression levels of some signaling molecules are elevated during aging. For example, a study on the IMR-90 cell line revealed that the levels of transforming growth factor beta 1 start to increase after incubation with H_2_O_2_, which is a method for the induction of cellular senescence in vitro, and this results in an increase in senescence-associated β-galactosidase (SA-β-GAL) activity and in the expression levels of some senescence-associated genes [24]. Another signaling molecule that is elevated during aging, according to many studies, is AMPK, which is responsible for cellular and organismal metabolism [25].

The clinical features of aged skin, apart from skin thickness and wrinkles, include xerosis, laxity and the occurrence of benign neoplasms. These features are accompanied by changes in the histology of the skin. Briefly, in the epidermis, there is a decrease in the number of melanocytes and Langerhans cells. Moreover, there is a reduction in the dermal–epidermal junction, which prevents the exchange of factors between these two compartments [26]. In the dermis, a decline in the dermal volume and in the number of blood vessels has been reported during skin aging [27]. Additionally, the levels of fat tissue start to run out and accumulate in pockets [28,29]. Furthermore, with age, there is a loss in bone mass, resulting in wrinkles and sagging [30]. These are some of the characteristics of skin aging, but they can also appear during the extrinsic aging process.

#### 1.2.2. Extrinsic Aging

Extrinsic skin aging is linked to lifestyle and results from exposure to several external factors, such as stress, ionizing irradiation, alcohol, environmental pollution, tobacco smoke and UV radiation. Due to its constant contact with the environment, the skin is more susceptible to extrinsic aging [8,31,32,33]. Among these factors, the main cause of extrinsic aging is UV radiation (also known as photoaging) [34,35]. Although UVC (100–280 nm) is mainly absorbed by the ozone layer, UVA (320–400 nm) and UVB (280–320 nm) are both responsible for alterations in the skin [35]. UVA is absorbed by both the epidermis and dermis and induces damage in the connective tissue of the dermis. Moreover, UVA increases the levels of ROS, which results in the activation of cell surface receptors for epidermal growth factor (EGF), insulin or keratinocyte growth factor (KGF); damages the lipids of the membranes; and causes DNA mutagenesis. Also, it is known that UVA is the main cause of photoaging [35,36,37,38]. On the other hand, UVB penetrates only the layer of the epidermis and can cause sunburn, tanning and photocarcinogenesis [8,35]. UVB induces DNA damage in keratinocytes and melanocytes of the epidermis and promotes the formation of thymidine dimers. The inability to resolve or repair thymidine dimers may lead, over time, to the accumulation of mutations [38]. Furthermore, exposure to UVB leads to the production of cyclobutane pyrimidine dimers (CPDs), which result in inflammatory responses, suppression of the immune system, induction of mutations or skin cancer [39,40]. Some clinical signs of photoaged skin include wrinkles, dryness, loss of elasticity, impaired wound healing, telangiectasia and formation of purpura [41]. 

The second most important factor that can cause extrinsic aging is cigarette smoking [42,43]. Smoking is linked to the dysfunction of many signaling pathways, such as the Tumor Necrosis Factor signaling pathway or Janus kinase signal transducer and activator of transcription (JAK-STAT) pathway. Moreover, it affects genome stability through telomere shortening [44,45]. It has been shown that, in smokers’ skin, the levels of matrix metalloproteinase 1 (MMP-1) mRNA are elevated, which leads to extracellular matrix breakdown [8]. The characteristics of aged skin, after years of smoking, include facial wrinkling, mainly around the mouth and eyes, and hyperpigmentation of the oral mucosa [43,46,47,48]. Aside from active smoking, passive smoking can also have detrimental effects. A study by Percoco et al. demonstrated that exposing human living skin explants to smoke results in an alteration in the skin surface physicochemistry. This alteration changes how the skin interacts with the environment. Additionally, the study found that smoke exposure raises the pH of the skin and disrupts its function as a natural barrier [49].

**Figure 1:**
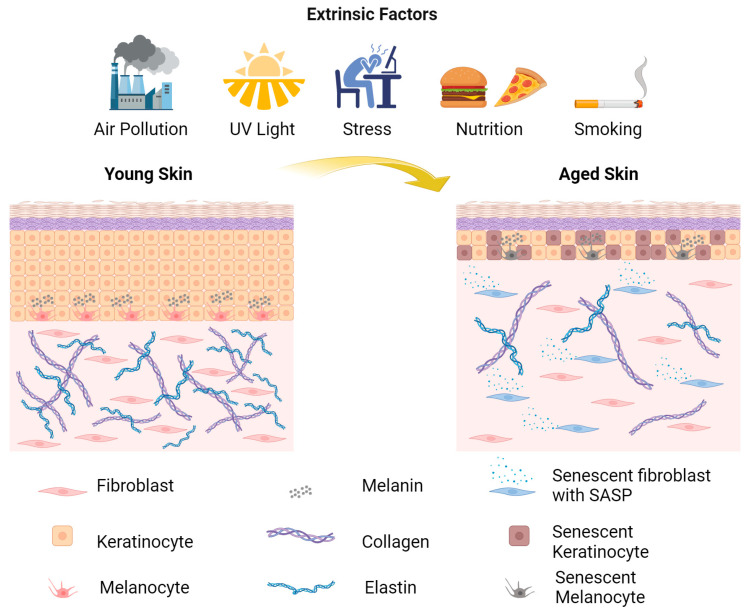
Schematic representation of extrinsic factors that can cause skin aging [8] (created with BioRender.com).

Particulate matter affects human skin, contributing to skin aging and the formation of wrinkles. Depending on the dose to which humans are exposed, it can induce cytotoxicity and increased expression of IL-1a. A study by Patatian et al. demonstrated that when human skin explants are exposed to a mixture of air pollutants, these pollutants penetrate the deepest epidermal layers, alter the gene expression profile and significantly increase the levels of extracellular vesicles in the stratum spinosum, indicating extensive cell communication [8,50,51,52,53]. A major air pollutant produced by diesel engines is diesel particulate matter, which induces ROS production and apoptosis in human dermal keratinocytes and elevates the expression of MMP-1 and MMP-3 in human dermal fibroblasts [54]. Moreover, nutrition plays an important role in the appearance of the skin, since a diet rich in antioxidants, with a high intake of vitamin C and less alcohol, can delay skin aging and the appearance of wrinkles [55,56,57]. Among the factors that can cause extrinsic skin aging are stress, which can interrupt skin integrity, sleep deprivation, which affects the appearance of the skin and elevated temperature of human skin, can increase the expression of MMP-1 [58,59]. Some of the extrinsic factors causing skin aging in the context of the skin aging exposome are represented in Figure 1 [8,58,59]. 

#### 1.2.3. Clinical Aspects of Skin Aging

As mentioned above, the most important clinical alterations seen in aged skin are the appearance of wrinkles, decreased skin thickness (atrophy), reduced elasticity and density, and loss of skin tone accompanied by a sallow color. Elderly skin shows reduced lipid content and decreased sebaceous and sweat gland secretion. These changes result in dryness of the skin (xerosis), with a tendency for irritation and erythema, contributing to skin barrier disruption. Capillary degeneration and decreased vascular content lead to dysfunctions in circulation and thermoregulation. Aged skin is susceptible to environmental injury, and, consequently, wound healing is delayed [60]. Dermatoporosis is a particular form of skin aging characterized by skin atrophy, senile purpura and pseudoscars (see below). There are several age-dependent skin diseases such as bullous pemphigoid, herpes zoster and erysipelas. The intrinsic and extrinsic skin aging process can also induce benign hyperproliferative, precancerous or malignant lesions, such as seborrheic keratosis, senile hemangioma, solar lentigo, actinic keratosis, basal cell carcinoma and squamous cell carcinoma.

#### 1.2.4. Histopathological and Ultrastructural Features of Intrinsic and Extrinsic Skin Aging

The thickness of the epidermis, during aging, is linked with a decrease in the number of dendritic cells and melanocytes. Consequently, the lower number of melanocytes affects the role of the epidermis as a protective barrier against UV radiation [61]. Moreover, during aging, there is a decrease in the number and secretion of sweat glands but not in their morphology, resulting in impaired thermoregulation in the human body [61]. Additionally, as skin ages, keratinocytes alter their shape and change from actively dividing cells to non-dividing ones [62]. Fragmented collagen fibers are present in aged skin and contribute to reduced collagen synthesis, possibly due to insufficient mechanical tension needed by fibroblasts to produce collagen [63]. Furthermore, the number of elastic fibers decreases in elderly people, and the density of hair follicles is reduced, without any change in their morphology [64,65]. A biopsy of a wrinkle shows a thinner stratum spinosum at the bottom of the wrinkle than on the wrinkle flanks, and the wrinkle cavity is often filled by a horny plug, well characterized by scanning electron microscopy [66].

On the other hand, extrinsic aging has the most dramatic effects on the skin. In more detail, photoaging induces an increase in the number of mast cells, histiocytes and fibroblasts. In addition to their increased number, these cells also exhibit abnormal morphology; for example, fibroblasts appear elongated and collapsed [64]. Furthermore, during extrinsic aging, there is an increase in the expression of specific proteases in the dermis, which affects the expression of collagen and elastin. Consequently, photoaged skin shows the accumulation of abnormal elastic tissue called elastosis. Ultrastructural analysis by transmission electron microscopy reveals the fragmentation of collagen fibrils, which are replaced by an amorphous elastotic material in aged and photoaged skin [67]. Additionally, specific kinases are upregulated, leading to the transcription of molecules such MMPs, which degrade the ECM [64,67]. Air pollution and cigarette smoke, which are two factors that promote extrinsic aging, induce the expression of MMPs and proinflammatory cytokines, which are responsible for the remodeling of the skin [64].

It should be noted that, irrespective of the cause of skin aging, there are some alterations in the dermal–epidermal junction. The dermal–epidermal junction, composed of a network of extracellular matrix macromolecules, plays a crucial role in maintaining structural integrity and regulating the cellular microenvironment [5,68]. Aging significantly impacts the structure and function of this junction, leading to alterations in skin physiology. In aged skin, the dermal–epidermal junction becomes flattened, resulting in the loss of epidermal ridges and dermal papillary projections. This reduced contact surface between the epidermis and dermis contributes to skin fragility and hampers the exchange of nutrients and oxygen. Furthermore, aging is associated with decreased expression levels of key components of the dermal–epidermal junction, including laminin-332, collagen IV, collagen VII and collagen XVII [5].

#### 1.2.5. Dermatoporosis: A Particular Form of Skin Aging

During aging, a gradual loss of hyaluronate (HA), the major component of the ECM that functions as a viscoelastic system between the epidermis and dermis, can be observed. This leads to extreme fragility of the skin and many complications such as lacerations, nonhealing ulcers and dissecting hematomas. We proposed, in 2007, the term “dermatoporosis” to cover different characteristics of a chronic cutaneous insufficiency syndrome. This new dimension of skin aging extends beyond cosmetics and appearance, aiming to understand its molecular mechanisms and develop preventive or therapeutic strategies for what turned out to be a prevalent skin condition recognized by the European Academy of Dermatology and Venereology [69,70,71,72,73,74,75]. Morphological markers of dermatoporosis include skin atrophy, characterized by wrinkles, senile purpura and pseudoscars. The first clinical signs of dermatoporosis are seen after the age of 40 and as wrinkles and appearance modifications; however, classical morphological markers of skin fragility develop after 60 years. Dermatoporosis usually starts with skin atrophy. Other signs, such as senile purpura, pseudoscars and superficial excoriations, may follow as the condition advances [2,17]. The topography of dermatoporosis indicates the role of ultraviolet irradiation in its etiology: the posterior side of forearms, dorsum of hands, presternal area, scalp and pretibial zones. Dermatoporosis has been proposed to have two forms: primary dermatoporosis, the most common type, resulting from chronological aging and long-term unprotected sun exposure, and secondary dermatoporosis, due to the chronic use of topical and/or systemic corticosteroids. Four stages of dermatoporosis have been proposed [76]: Stage I: in this stage, we find the above-mentioned morphological markers of dermatoporosis; this is the most common stage of dermatoporosis. Stage IIa: localized and small superficial lacerations (<3 cm) due to skin fragility. Stage IIb: larger lacerations (>3 cm). Stage IIIa: superficial hematomas. Stage IIIb: deep dissecting hematomas without skin necrosis. Stage IV: large areas of skin necrosis with potential lethal complications. Dermatoporosis is frequent among the elderly population. Resulting in skin tears and deep dissecting hematoma, it can lead to significant morbidity and mortality. Better knowledge and treatment of this pathology should be spread among healthcare professionals to better prevent and treat the resulting lesions.

Many different factors are responsible for the fragility of skin during dermatoporosis, such as alterations in the viscoelasticity of the skin or the upregulation of MMPs in combination with the downregulation of their inhibitors, which degrade the ECM. In addition, studies have shown that a reduction in the expression of HA and its receptor CD44 can lead to epidermal atrophy [69]. CD44 is the main receptor of HA, and it regulates the proliferation of keratinocytes. As a result, defects in HA and CD44 play an important role in skin atrophy and the development of dermatoporosis [77,78,79,80]. This decrease in HA and CD44 levels can also be observed due to UVA or UVB exposure or as a result of the topical use of corticosteroids [81].

### 1.3. Cellular Senescence

During aging, senescent cells accumulate in the skin, disrupting its normal function and structure [82,83,84]. Cellular senescence was first characterized as a hallmark of aging by Hayflick and Moorhead (1961). They reported that cellular senescence contributes to a decline in tissue functionality by impeding tissue repair and regeneration [83,84,85,86,87]. Several stressors can cause senescence in cells, such as telomere shortening, mitogenic signals, oncogenic activation, irradiation, stress, mitochondrial dysfunction, inflammation, nutrient deprivation, etc. Given that cellular senescence is a heterogenous process, various markers can be used to characterize these cells [88,89,90,91].

Despite multiple molecular, cellular and phenotypic changes (such as enlarged and flattened morphology) occurring in senescent cells, these cells remain metabolically active, show resistance to apoptosis and are characterized by an irreversible cell-cycle arrest, mediated by p21 and p16INK4a. Senescent cells express higher levels of DNA damage markers, such as γ-H2AX as well as cell cycle regulators such as p53, p21 and p16INK4a, as opposed to normal cells [34,92,93]. Moreover, senescent cells exhibit a senescence-associated secretory phenotype (SASP), which includes the secretion of high levels of proinflammatory cytokines, chemokines, growth modulators, angiogenic factors and matrix metalloproteinases [84,94]. Table 1 summarizes the main SASP factors, which are released by senescent cells (cellular senescence) and the types of cells that express them. These factors can promote senescence in neighboring normal cells in a paracrine manner. In addition, SASPs may activate immune responses, contribute to chronic inflammation (inflammaging) and have been reported to promote tumorigenesis [84,85,87,95,96,97,98,99,100,101]. Recently, it has been shown that circulating SASPs are an indicator of age and are a medical risk in humans [102]. The expression of SASPs is regulated by NF-κB and p38 MAPK signaling pathways, while their recycling occurs in an autocrine way [103,104].

In addition to the aforementioned SASP factors, extracellular vesicles (EVs) are also associated with senescence. Specifically, EVs are cell-derived nanoparticles released by almost all cells, carrying bioactive materials such as nucleic acids, lipids and proteins to other cells. They are involved in regulating intercellular communication and various processes, including immune regulation, cell growth and differentiation [105,106]. Evidence suggests that EVs significantly influence aging, with senescent cells releasing more EVs that can induce senescence in neighboring cells [106,107]. Beyond their role in promoting senescence, EVs hold potential as therapeutic agents; they can deliver anti-aging drugs or serve as biomarkers, with their removal potentially offering protection against age-related diseases [107,108].

Furthermore, senescent cells are characterized by strong SA-β-GAL activity, a marker that distinguishes senescent cells from quiescent and postmitotic cells [109]. It should be mentioned that, in contrast to cellular senescence, quiescence is a temporary arrest of the cell cycle, which can be reversed under certain circumstances. However, some studies have reported that senescent cells, mainly in tumors, can re-enter the cell cycle [110,111,112].

It is important to note that aging and cellular senescence are not synonymous terms. Senescence can occur at any stage of life, with beneficial effects such as the maintenance of tissue homeostasis and wound healing [113,114]. In addition, during embryogenesis, senescent cells contribute to tissue development, and in later life stages, they can play an important role in tissue repair and tumor suppression [91,114,115]. Controlled and regulated senescence is favorable for tissue homeostasis, in contrast to senescent cells expressing SASPs, which may inhibit tissue repair and regeneration, thereby contributing to aging [83,84]. Moreover, cellular senescence can result in cell-cycle arrest and prevent the carcinogenic mutations to pass through other cells and induce the clearance from the immune system. Nevertheless, there are some pre-clinical and clinical data that show a strong positive correlation between a reduction in senescent cells and functional improvements in the skin [116].

#### 1.3.1. Senescence in Skin Cells

Fibroblasts are the most abundant cell type in the dermis, and their senescence is a primary driver of skin aging [35]. Many intrinsic and extrinsic factors can promote the shortening of telomeres, mitochondrial dysfunction and cell-cycle arrest in fibroblasts, leading to their senescence. Senescence in fibroblasts results in the expression of SASP factors such as interleukins, MMPs and other chemokines, accompanied by an increase in the levels of p16INK4a, p21, p53 and SA-β-gal [109,117,118]. Elevated MMP expression leads to a reduction in collagen and the formation of wrinkles [119,120,121]. Mechanistically, during chronological aging, the expression levels of TGFβRII are reduced, thereby compromising TGFβ signaling [122]. Consequently, MMPs, which are normally downregulated through the TGF-β signaling pathway, become upregulated and lead to the degradation of collagen, promoting a reduction in skin thickness [123].

During aging, keratinocytes from the basal layer of the epidermis exhibit upregulated expression of MMPs (such as MMP-1), which induces downregulated expression of ECM components (such as collagen) and reduced proliferation rates [124,125]. Furthermore, UV exposure can induce the generation of ROS in keratinocytes and trigger the ERK, p38 and JNK signaling pathways, leading to activation of the transcription factor AP-1 and enhancing the expression of MMPs. Apart from MMPs, proinflammatory cytokines are highly expressed in senescent keratinocytes [126]. Previous studies have indicated that UVB exposure increases the levels of p16INK4a, p21 and p53 in keratinocytes, along with enhanced SA-β-GAL activity. On the other hand, samples from photoprotected skin areas revealed that melanocytes are predominantly p16-positive [126,127]. Moreover, an increase in the number of p16-positive cells has also been observed in the epidermis of age-related pathologies, such as dermatoporosis [128].

The populations of melanocytes decline during skin aging, leading to reduced melanin production and decreased protection from the harmful effects of UVR [41]. Senescent melanocytes are subjected to morphological changes such as increased size and expression of inflammaging markers. There is also a change in the size of melanosomes and an elongation of their dendrites [129]. Moreover, it is proven that melanocytes are the primary epidermal cells expressing p16INK4a during aging, especially in parts of the skin which are photoprotected. Their SASP induces in the neighboring cells, such as keratinocytes, a telomere dysfunction and prevents their proliferation [127,130].

The skin provides a defense for our body, through several types of immune cells, such as Langerhans cells, dendritic cells, macrophages, monocytes and T cells [131]. During aging, the functionality of the immune cells of the skin is decreased, and this results in an increased risk of skin infections and cancer [132]. For example, Langerhans cells show decreased proliferation and migration rates, combined with reduced expression of antimicrobial peptides, avoiding the protective barrier of the skin against microorganisms. Moreover, there is a reduction in the levels of IL-1β, which is linked to age-related changes in immune function, due to either overall reduced transcription or a decreased number of Langerhans cells, as they are, especially in mice, the main source of IL-1β. In addition to the reduction in the number of immune cells in the skin, an increase in specific populations may have adverse effects. Previous studies have reported that samples from photoaged skin show increased numbers of monocytes and macrophages, which express MMPs and ROS, leading to degradation of the ECM and chronic inflammation [133,134].

##### Methods for Induction of Cellular Senescence

Despite the scientific knowledge gained over the past few years in the field of aging, the mechanisms of cellular senescence and their implication in age-related diseases are not fully understood. The development of assays to study senescence in vitro are of major importance, in order to bridge the gap in knowledge regarding the molecular pathways that are implicated in senescence [135].

Regarding cellular models for studying senescence, the use of different cell lines is quite common. One of the most widely used cell lines is the normal human epidermal keratinocytes (NHEKs), which display the same biochemical properties as skin keratinocytes. However, one limitation is that primary keratinocytes have a high heterogenous population, as they composed of stem cells and differentiated cells [136]. Another cell type that is extensively used is the HaCaT cell line, which can divide infinitely. It is easy to handle and resembles the characteristics of human keratinocytes [137,138]. A limitation of using HaCaT cells is their poor response to Ca^2+^ compared to NHEKs and their abnormal expression of proteins like filaggrin and loricrin [139,140]. Other than these cell types, fibroblasts and melanocytes can also be used for the study of cellular senescence in vitro. Except from 2D cell-line-based assays, 3D in vitro skin models containing all viable and nonviable cell layers and different cell types are commonly used now. Cultivation is easy, with the probability of multiplexing, while it may limit or even replace the use of animal models. Despite their advantages, 3D skin models still have some limitations, as they are composed of only one cell type, and there are no collagens, fibroblasts, blood vessels, melanocytes, Langerhans cells and leukocytes [141].

One of the most well-studied methods to induce senescence in vitro is the use of ionizing or ultraviolet irradiation to induce DNA damage. Apart from different types of irradiation, chemotherapeutic drugs and crosslinking agents, such as doxorubicin, can also promote cellular senescence in vitro through the transcription and replication of DNA damage [142,143,144]. In addition, the induction of oxidative stress through exposure to hydrogen peroxide (H_2_O_2_) leads the cells to a non-proliferative state, which is known as stress-induced premature senescence. Moreover, histone deacetylase inhibitors and DNA demethylating agents can be used in vitro in order to promote senescence in normal cells, since the expression of genes implicated in the induction of senescence is controlled by histone acetylation and DNA methylation [145,146]. Several methods and assays have been developed to monitor cellular senescence, such as the detection of β-galactosidase (β-GAL) activity or immunodetection of the phosphorylated form of the histone variant H2AX at serine 139, a sensitive marker for DNA double-strand breaks. Immunoblotting for the senescence-associated proteins, such as p16, p21, p53 or the immuno-detection of SASPs through ELISA, could be applied [147].

#### 1.3.2. Investigation of Aging In Vivo

Apart from the cell-based assays previously described, transgenic mice have been developed in order to study the aging process and the role of p16INK4a protein in vivo. Many different mouse models are used to investigate the role of senescent cells and the impact of their removal in aging and age-related diseases [148,149]. The p16-3MR mice express a trimodality reporter fusion protein, which contains domains of Renilla luciferase (rLUC), monomeric red fluorescent protein (mRFP) and herpes simplex virus thymidine kinase (HSV-TK) under the control of an artificial p16 promoter. This mouse model allows for the elimination of senescent cells by using ganciclovir, which is converted into a toxic DNA chain terminator by HSV-TK [114]. In the INK-ATTAC mouse model, an FKBP-Caspase 8 fusion protein is expressed, fused with an enhanced green fluorescent protein (eGFP) under the control of a p16 promoter. The drug AP20187 induces the dimerization of FKBP-Caspase 8 fusion protein that is transcriptionally active in senescent cells [150]. Another mouse model that is used to study aging is BubR1^H/H^. It is known that a reduction in BubR1 protein causes chromosome aneuploidy and senescence [151,152]. BubR1^H/H^ mice express less BubR1 protein than wild-type mice and exhibit a variety of age-related phenotypes. During aging, p16 protein is accumulated in many tissues, and its elimination can delay many defects that are linked with age. By crossing BubR1 with INK-ATTAC mice, a progeroid mouse model is created. In this mouse model, the elimination of p16+ cells can be achieved by using the drug AP20187 [150,151,152]. Sod1^−/−^ is also a progeroid mouse model, which exhibits increased oxidative DNA damage, elevated p16 and p21 expression and many changes that also appear during human aging, such as hearing loss, cataracts, epidermal thinning, etc. [153,154,155]. These mouse models are widely used in studies for aging, but it is not still clear if the elimination of senescent cells or their SASP is responsible for the positive effects in different age-related diseases [87].

### 1.4. Senescent Cells: A Novel Therapeutic Target for Skin Aging

Many efforts are invested in discovering therapeutics that will directly target senescent cells or eliminate the SASP, which induce senescence in a paracrine way, in order to prevent age-related diseases and increase the healthspan [156]. The clearance of p16Ink4a-expressing cells in BubR1-hypomorphic progeroid mice delays aging-associated disorders [150,157], and the results of other studies suggested that elimination or weakening of the function of senescent cells may be a promising approach for the modulation of fundamental aging processes. These strategies are collectively named ‘senotherapies’ [87]. There are two kinds of senotherapeutics: senolytics, which induce senolysis in senescent cells by facilitating apoptosis due to their own SASP, and senomorphics, which attenuate their pathological proinflammatory secretory phenotype to cause senostasis [158]. Each senotherapeutic modality has various advantages and disadvantages. Many senolytic agents including synthetic small molecules and peptides have been developed for the in vitro and in vivo elimination of senescent cells [159,160]. There is also some amount of pre-clinical and clinical data showing a strong positive correlation between a reduction in senescent cells and functional improvements in the skin [116]. Moreover, the potential senotherapeutic effect of different compounds in skin aging has been suggested in different studies. For example, combining Dasatinib and Quercetin reduces the expression of p16 and p21 in the human epidermis, whereas rapamycin has been found to reduce senescent cells in human skin [161,162,163,164,165].

Senescent cells avoid apoptosis through the induction of prosurvival pathways. For this reason, inhibitors of prosurvival pathways may specifically target senescent cells [166]. The first senolytic drugs identified, which act through inhibition of these prosurvival pathways, were Dasatinib and Quercetin [101,167]. Dasatinib is an inhibitor of multiple tyrosine kinases and is used as an anticancer drug, whereas Quercetin is a flavonol [101,159]. Treatment with these drugs in a murine model showed a reduction in the viability of senescent cells, with the combination of both drugs being more effective, as opposed to single treatments. In more detail, in aged mice, there was a decrease in the number of senescent mouse embryonic fibroblasts (MEFs) and in the p16 expression in fat tissue and liver. Furthermore, the expression of anti-apoptotic regulator PAI-2 was reduced, and the prosurvival networks of senescent cells were eliminated. Moreover, the combined treatment decreased the expression of p16 in muscle cells and the number of cells, which were positive for SA-β-GAL following irradiation-induced senescence [101]. Importantly, the combined administration of Dasatinib and Quercetin reduced the expression of p16 and SA-β-gal in a phase I clinical trial in patients with diabetic kidney disease and idiopathic pulmonary disease [167,168]. Several other studies have shown that the Dasatinib and Quercetin combination is highly specific in targeting senescent cells, increasing the survival of mice and improving their healthspan [167,169]. Moreover, systemic treatment of patients with systemic sclerosis with Dasatinib for 169 days has shown a decrease in the expression of SASP factors and other age-related genes that belong to pathways, such as hypoxia, the TNF-alpha pathway, the p53 pathway and the inflammatory response signaling pathway, when comparing clinical improvers with non-improvers [170]. While a reduction in senescence markers in skin after systemic treatment with the senolytic combination Dasatinib and Quercetin has been demonstrated in one clinical study [167], clinical studies showing improvements in skin function after senolytic intervention are still missing. Similarly, there is only a single preclinical study showing relief of UV damage in mouse skin following topical senolytic (fisetin) application [171].

Senescent cells develop resistance to apoptosis through the upregulation of negative modulators, such as members of the B-cell lymphoma 2 (BCL-2). For this reason, inhibitors of the BCL-2 family of proteins can be used as senolytic drugs [172,173]. ABT-263 (known as navitoclax) and ABT-737 are the most frequently used BCL-2 inhibitors in vivo, which foster senescent cells to initiate apoptosis. However, these drugs did not show high potency, and further studies should be performed in the future in order to assess them [174]. Additionally, during senescence, forkhead box protein O4 (FOXO4) binds to p53. The administration of FOXO4 inhibitors can interfere with FOXO4 binding to p53 and lead to the locomotion of p53 and subsequent release of cytochrome C from the mitochondria and the apoptosis of senescent cells [175]. Ginsenoside Rb1, an ingredient of traditional Chinese medicine, is known to have several positive effects. A study from Yu et al. showed that ginsenoside prevents the aging process in mice by regulating cell-cycle progression but has no effect on the expression of SASP factors [176].

Another senolytic molecule known for its physiological functions, including sleep, circadian rhythms and neuroendocrine actions, is melatonin. The levels of melatonin decrease with age, but both in vivo and in vitro studies have shown anti-aging properties. In more detail, it increases the activity of DNA repair enzymes; it prevents apoptosis, protects the DNA from UV irradiation and protects from inflammaging [177,178,179]. A recent study showed that melatonin also has senolytic effects ex vivo, as it can downregulate the mTORC1 pathway and the expression of MMP-1 and upregulates the expression of molecules which are important for skin rejuvenation [180].

**Figure 2:**
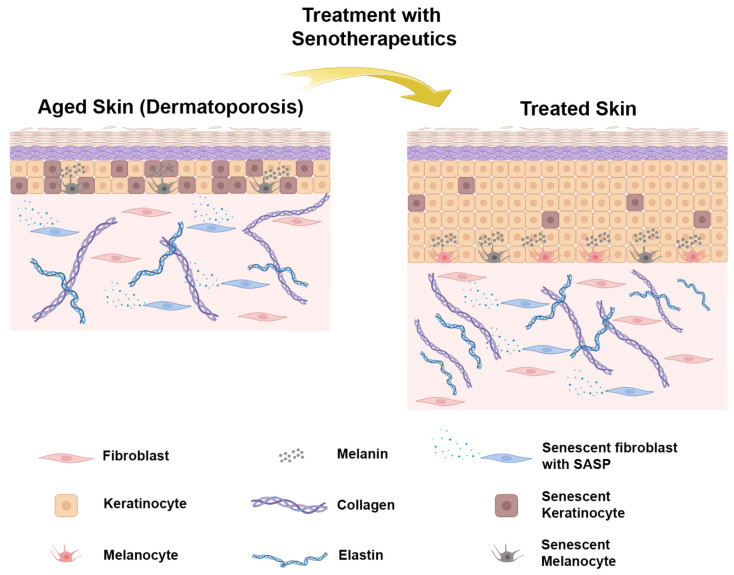
Schematic representation of skin with dermatoporosis, before and after the treatment with senolytics (created with BioRender.com).

As mentioned before, CD44 is the main cell surface receptor for HA and, in combination with other molecules, forms the hyalurosome. Suppression of CD44 leads to skin atrophy [80], but a combination of retinaldehyde (RAL) with HA fragments (HAFi) induces skin hyperplasia [181]. Topical application of RAL and HAFi for 1 month significantly reduced the number of p16Ink4a-positive cells in the epidermis and dermis in dermatoporosis patients, which also showed a significant clinical improvement with an increase in skin thickness, showing a senotherapeutic effect [181,182] (Figure 2).

Apart from directly targeting senescent cells, another strategy is to block the production and secretion of SASP factors, with agents known as senomorphics [158]. It is known that the mTOR pathway promotes the expression of SASP factors. In more detail, mTOR is a serine/threonine kinase that regulates cellular growth and metabolism. It is evolutionary conserved and consists of two different complexes, mTORC1 and mTORC2, which differ functionally and structurally [183,184]. As many of the hallmarks of aging are affected by mTOR, the depletion of mTORC1 in non-vertebrates enhances longevity [185]. Rapamycin is used as an inhibitor of the mTOR pathway and has been reported to delay aging and extend lifespan in mice through a reduction in p16 protein levels and an increase in the expression of collagen VII [186,187,188]. The disadvantage of this therapy is that its long-term effects on people’s healthspan are not fully clarified, and the mechanism of mTORC1 downregulation is not fully understood [189]. Another compound with a senomorphic effect is metformin, which modulates the NF-κΒ signaling pathway and reduces the secretion of SASP [190,191]. Furthermore, a number of monoclonal antibodies against components of the SASP or their receptors, such as IL-6, IL-1α, IL-1β and TNF, have been described to harbor senomorphic effects [192,193,194,195].

Resveratrol is another agent that may have both beneficial and detrimental effects during the aging process. At low concentrations, it can act as a senomorphic and can suppress cellular senescence and SASPs, but at higher concentrations, it can promote senescence and/or cell death. Furthermore, in vivo studies have shown that, in mice with high-fat diets, resveratrol can extend their lifespan, but it does not have the same effect in mice with standard diets [196,197]. Additionally, procyanidin C1 (PCC1), a compound found in grape seed extract flavonoids, has both senolytic and senomorphic effects. At low concentrations, it can eliminate the SASP factors, whereas at higher concentrations, it can selectively kill senescent cells through the induction of apoptosis, according to in vivo studies [198,199].

Currently, senolytics seem to be a better anti-senescence strategy compared to senomorphics, since they can directly target senescent cells, and there is no need for the continuous administration of inhibitors for the expression of SASP factors. Moreover, by using senolytics, there is less risk for the generation of mutations in senescent cells, which will favor tumorigenesis [87].

While current senotherapies appear promising, they are associated with various side effects, and there are no established guidelines for their timing and dosage. For example, the systemic administration of senolytics may increase the risk of cirrhosis in patients with liver fibrosis or hinder wound healing, since senescent cells are crucial for this process. Consequently, further research is needed to discover new senotherapies or to improve existing ones to reduce these side effects [200,201]. These novel therapies would modulate the senescence secretome by selectively targeting SASPs, such as interleukines, chemokines, growth factors or MMPs (Table 2). In addition, further studies should be performed in order to determine whether prolonged treatment with senolytics or senomorphics or their administration in advanced age stages may show toxicity.

### 1.5. Discussion

Skin senescence occurs due to exposure to several environmental factors or with age onset. Since the skin is a tissue which communicates with many different organs, SASPs, which are secreted from skin senescent cells, can direct senescence in other tissues/organs [9]. Given the nature of senescence and the high heterogeneity of SASPs in aged tissues, the discovery of selective biomarkers is challenging [34]. Therefore, a multilevel and high-throughput approach is required. Indeed, Gorgoulis et al. proposed a multi-marker approach consisting of a three-step workflow in order to detect senescent cells in a given sample. The first step includes screening for SA-β-gal activity. The second step is the verification of senescence with co-staining for frequently observed markers of senescent cells, like p16INK4a, p21 and some other SASP factors. The last step includes the identification of markers that are expressed in specific types of senescence [91]. Moreover, single-cell analysis can provide more insight about the presence of senescent cells in specific tissues and their role in tissue function as well as in the aging process [91,214].

Until now, the selective removal of senescent cells or the modulation of SASP factors has been assessed by using specific drugs (senolytics or senomorphics), which can prevent their accumulation and suppress age-related pathologies. It should be noted that these drugs should exhibit high potency in order to prevent senescent cells from acquiring drug resistance. This phenomenon may occur through the acquisition of mutations, which may favor the cell-cycle re-entry of a subpopulation of senescent cells, leading to the generation of aggressive clones [91,111,160,215]. To this end, further research is required to investigate potential interactions between the pathways that are responsible for SASP expression. Other future objectives include the identification of the whole repertoire of SASP factors, the investigation of their role in age-related diseases and the identification of cell subpopulations that undergo senescence [216,217,218]. Studies using the INK-ATTAC mouse model have shown that the elimination of p16INK4a-positive senescent cells delays age-related diseases and improves the healthy lifespan, as senescent cells promote tumor progression and impairment in certain organs, and there were no adverse effects from their removal [150,157]. According to studies, the use of senolytic agents is a promising approach for age-related pathologies, as they eliminate the appearance of senescent cells, but we should mention that the impact of senolytic-based therapies in humans is still unknown [182].

In conclusion, skin aging results from various internal and external factors that induce cellular senescence. This process significantly affects people’s lives, being associated with different pathologies and promoting senescence in other tissues through the secretion of SASPs. Recently, senotherapies have been developed to eliminate senescent cells or the factors they secrete, but it remains unclear whether these therapies have any consequences for humans. To this end, further research should be performed in order to elucidate the role of senescent cells and SASP in organismal aging, as it is known that they are not synonymous and shed light on the impact of their removal in humans as well as the effect of skin aging in the aging process of other tissues and organs. For this reason, our group is currently working on the identification of some molecules that can reduce senescent cells and SASP factors in aged mouse and human skin. The therapeutic removal of p16Ink4a-positive cells that accumulate during senescence may be an attractive approach to reverse skin aging and lead to discoveries of novel targeted therapy strategies.

## Figures and Tables

**Table 1:** Summary of some of the SASP factors that are expressed during aging and the type of cells that express each SASP factor. Data are mainly based on Zorina et al., 2022 [88].

Category	SASP Factors	Cell Types (That Mainly Express These SASP)
Interleukins	IL-1, IL-1b, IL-6, IL-7, IL-13, IL-15	Fibroblasts, Keratinocytes & Melanocytes
Chemokines	IL-8, GRO-a, GRO-b, GRO-g, MCP-2, MCP-4, MIP-1a, MIP-3a, HCC-4, eotaxin-1, eotaxin-3	Fibroblasts, Keratinocytes and Melanocytes
Insoluble factors	Fibronectin, collagens, laminin	Fibroblasts & Keratinocytes
Inflammatory molecules	TGFβ, GM-CSE, G-CSE, IFN-g, BCL, MIF	Keratinocytes. TGFβ and G-CSE are also secreted from fibroblasts, GM-CSE from macrophages, IFN-g from T-cells, BCL from lymphocytes and MIF from macrophages
Non-protein molecules	ROS, NOS, miRNAs	Keratinocytes and Fibroblasts. ROS are also secreted from melanocytes, NOS from endothelial cells and miRNAs from melanocytes
Growth factors and regulators	EGF, bFGF, HGF, KGF, VEGF, SCF, SDF-1, PIGF, NGF	Fibroblasts and Keratinocytes
Receptors and ligands	ICAM-1, ICAM-3, OPG, sTNFRI, sTNFRII, TRAIL-R3, Fas, uPAR, EGFR	Fibroblasts and Keratinocytes
Proteases and regulators	MMP1, MMP3, MMP10, MMP12, MMP13, MMP14, TIMP1, TIMP2, PAI1, PAI2	Fibroblasts, Keratinocytes and Macrophages

**Table 2:** Summary of some of the senolytics and senomorphics that have been identified as well as their method of action.

Compound	Target	Disease	Effect	References
Dasatinib + Quercetin	Inhibition of prosurvival pathways	Idiopathic pulmonary fibrosis (IPF) Systemic sclerosis (SSc) Persistent physical dysfunction	Senolytics Reduce SASP factors in humans and the adipose tissue senescent cell burden and in mice reduce senescent cells	[167,169,170]
ABT-263 & ABT-737	Block the interaction of BCL-2, BCL-XL, BCL-W with BCL-2 homology domain containing proapoptotic proteins	DNA damage in lungs Aged epidermis	Senolytics Elimination of senescent cells in mice	[158,202,203]
FOXO4-DRI	Blocks the interaction of FOXO4 and p53	Aging	Senolytic Eliminates senescent human fibroblasts and chondrocytes in vitro and senescent cells in mice	[175,204]
Fisetin	Antioxidant	Progeroid syndrome Skin damage from UV exposure	Senolytic Decreases specific types of senescent cells in murine models and in human adipose tissue explants	[171,205]
Geldanamycin	HSP90 inhibitor	Aging	Senolytic In human cells and in mouse models delayed age-related co-morbidities	[206,207]
Panobinostat	Histone deacetylase inhibitor	Upper aerodigestive and lung malignancies	Senolytic In vitro kills senescent cells that accumulate during chemotherapy	[208]
Metformin	Prevention of oxidative stress	Stress-induced senescence of adipose derived stromal cells	Senomorphic In human cells decreases senescence and SASPs	[209]
Rapamycin	Target mTORC1	Cellular senescence	Senomorphic Reduces senescence and SASPs in cell lines from human, mouse and rat	[158]
SB203580	p38MAPK inhibitor	Senescence caused by irradiation	Senomorphic Reduces the secretion of SASP in mice	[210]
Procyanidin C1	Inhibition of SASP or induction of mitochondrial dysfunction	Aging and age-related diseases	Senolytic and senomorhic At low concentrations reduce the SASP, whereas in higher concentrations promotes the production of ROS	[198,199]
Rutin	Weakens the interaction between ATM and TRAF6	Cellular senescence	Senomorphic Reduces the expression of SASP	[211]
Niacinamide & Hyaluronic acid	Antioxidant Anti-inflammatory Immunomodulatory	Skin aging	Senomorphic Reduces the expression of SASP	[212]
Polyphenolic Flavonoids	Targeting of regulatory pathways (such as p38 signaling pathway, PI3K/Akt, mTOR and JAK/STAT) or the expression of transcription factors or directly SASP factors	Age-related diseases	Senomorphic Reduces the expression of SASP	[213]
